# Promoting Social Entrepreneurial Organizations: An Empirical Study of Teacher–Student Co-entrepreneurship

**DOI:** 10.3389/fpsyg.2020.01470

**Published:** 2020-07-23

**Authors:** Jinchen Du, Guanshuang Han, Zhaoxin Huang

**Affiliations:** ^1^School of Innovation and Entrepreneurship, Wenzhou Medical University, Wenzhou, China; ^2^College of Education, Zhejiang University, Hangzhou, China

**Keywords:** teacher–student co-entrepreneurship, collaborative co-entrepreneurship, employment co-entrepreneurship, cultivation model, social entrepreneurship organization

## Abstract

This paper explores the impact of different types of social entrepreneurship education cultivation models on students’ opinion on social entrepreneurship, defines and classifies the emerging social entrepreneurship cultivation models in colleges and universities, and confirms the performance difference between the teacher–student co-entrepreneurship (TSCE) cultivation model and the traditional cultivation model through quantitative research. Chi-square analysis and the structural equation model are used to verify the following two conclusions: in the process of promoting the acceptance of social entrepreneurship study, the TSCE cultivation model is more effective than the traditional cultivation model; and the collaborative co-entrepreneurship cultivation model is more conducive to the deepening of practical learning than the employment co-entrepreneurship cultivation model. The research results will help colleges and universities to redesign the training process of social entrepreneurship skills, and then provide help for college students to achieve more adequate entrepreneurial preparation.

## Introduction

China’s entrepreneurship education in universities has entered a stage of total restructuring (Liu, 2017). As a new research topic on entrepreneurship education in China, social entrepreneurship education has shown to perform well in solving multiple issues and bringing about social change (Nosratabadi et al., 2019; Wu et al., 2020). Social entrepreneurship education, as a new intersection of education and entrepreneurship in China, is still in its infancy. Different from the popularization of entrepreneurship education, many universities are still exploring its development path and cultivation model.

Nowadays, the development of social entrepreneurship education faces two challenges. From the theoretical level, there is no consensus on the clarification of basic concepts. For example, what level of quality should social entrepreneurs have, how should social entrepreneurs be trained (Abereijo, 2019), how should the effectiveness of social entrepreneurship education be inspected (Kee, 2019), and what are the differences or connections between social entrepreneurship education and business entrepreneurship education? These questions from the field of education and academia lack persuasive and generally accepted answers in Chinese higher education, which means social entrepreneurship education lacks effective references in the design of talent cultivation.

Secondly, from the practical level of social entrepreneurship education, there are several kinds of disconnection. The first is the gap between competition and launch (McAdam et al., 2016), which shows that although many social entrepreneurship teams can achieve competition results, they may not end up launching, or they fail to survive the baby or incubator stage.

The second kind is the disconnection between individuals and groups. In this kind of situation, the vision heterogeneity and utilitarian tendency of the members (Yu and Xiang, 2018) of the social entrepreneurship team causes core members to leave and personal interests to dominate thinking, which limits the development and expansion of social entrepreneurship organization (SEO). The third disconnection occurs in theory and practice. Due to the dispute between the abstract definition of theory and the conclusion, and the lack of real case references, students develop a social mission while they actually operate completely according to business enterprises in entrepreneurial practice. It makes their mission and actions disconnected.

In short, due to the short development time and immature level, social entrepreneurship education in today leads to low production and high consumption situation. Therefore, in order to improve the efficiency of social entrepreneurship education in colleges and universities, approaches related to cultivating social entrepreneurship talents need to be explored. This paper aimed to summarize practical experience and relate it to academic theory, providing references for social entrepreneurship education development.

According to our follow-up, interview, and investigation of social entrepreneurial teams in colleges and universities in the past year, we found that team members are more likely to show higher organizational dedication and organizational citizenship behavior when they have a shared vision of entrepreneurial goals. Moreover, when the team project is implemented, it may also show better business performance or social impact. And in this kind of team, the cooperation between teachers and students is very close. Some of these teachers are entrepreneurial mentors, while some are subject teachers. The relationship between teachers and students is similar to that between teachers and friends. Through the analysis of the social network of emotional networks and consulting networks, we found that there are frequent links between teachers and students in the two kinds of networks, with high cohesion and few isolated points. Therefore, we believe that a shared vision and deep cooperation between teachers and students may contribute to the high performance and organizational effectiveness of social entrepreneurial teams.

Based on this, we put forward the following two hypotheses: teacher–student co-entrepreneurship (TSCE) promotes SEO development and launch; and when teachers and students hold a common vision, social entrepreneurial teams are more likely to achieve success with their SEO.

## Literature Review

### Social Entrepreneurship’s Characteristics and Definition

Social Entrepreneurship (Dees, 1998), as a new organizational form, has the inherent advantages of promoting social change in the context of a market economy (Mair and Martí, 2006). It usually contains the following features: business operations, promoting public welfare, and being transformative in nature. Different from traditional business organization, SEO achieves the breakthrough in the value chain through innovation and makes itself obtain core competitiveness. Compared with previous social welfare institutions, the profitability of social entrepreneurship enables them to have a hematopoietic capacity and, to a certain extent, the ability for sustainable development. Focusing on acute social problems, social entrepreneurship faces the test of “three party failure” (market failure, government failure, and third sector failure) to seek new ways to demand integration and value expression. Its success will bring both commercial and social returns, and even change the market structure.

Based on the previous definitions of social entrepreneurship (Rashid, 2019), generalized definitions of social entrepreneurship refer to all organizational or non-organizational forms of social welfare activities that bring social value. In a narrow sense, social entrepreneurship refers to the establishment of social entrepreneurship enterprises in the corporate form by means of commercial means with the purpose of maximizing social value. This paper defines social entrepreneurship in a broad sense. Therefore, the objective event investigated in this paper is to establish social entrepreneurship studios, which is regarded as SEO.

**FIGURE 1:**
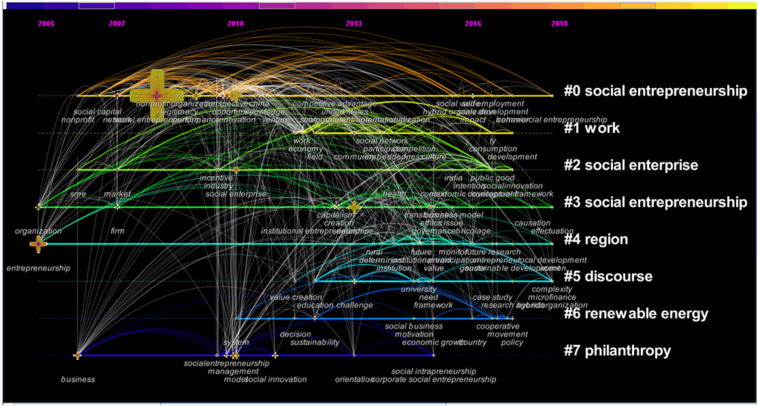
Development of the social entrepreneurship research theme (1998–2018).

According to a research map of social entrepreneurship, the research focus of social entrepreneurship in the past five years has shifted to public governance, college students, and higher education (see Figure 1). As a hot research topic, the social entrepreneurship of college students and the social entrepreneurship education of colleges and universities have attracted much attention. How to promote the acceptance of social entrepreneurship in colleges and universities, and what factors may affect the acceptance of social entrepreneurship in the current colleges’ incubation chain, are the focuses of this paper.

### Social Entrepreneurship’s Cultivation Model in Colleges and Universities

Based on previous research, the discussion of social entrepreneurship education on the cultivation of social entrepreneurship talents is gradually moving toward defining a clear role, scientific education, and multiple integration. The development of social entrepreneurship education is first reflected in the independence and perfection of cognition. Ashcraft et al. (2019) discussed the ownership and definition of social entrepreneurship curriculums in the early stage, and put forward three development paths of social entrepreneurship curriculum: social entrepreneurship curriculums under business schools, non-profit enterprise curriculums, and mixed social entrepreneurship curriculums. Later, based on the results of cross research, compared with the opening and enrollment of business entrepreneurship education courses in the same period, Solomon et al. (2019) concluded that nowadays college students have strong social entrepreneurship tendencies and a strong demand for their development status. In contrast, the supply of social entrepreneurship courses is insufficient. This process shows that social entrepreneurship education has gradually established its own educational mission and methods. However, some researchers also point out that the academic independence and academic value of social entrepreneurship education in underdeveloped regions are still under the shadow of business education (Waghid, 2019).

Secondly, the development of social entrepreneurship education is reflected in the exploration and discovery of educational techniques through the determination of researchers and the enrichment of social entrepreneurship education practices. Educators have developed many valuable conclusions on social entrepreneurship courses, teaching methods, and learning techniques. For example, in the study of social entrepreneurship learning and social entrepreneurship willingness, some scholars find that effective integration of students’ social capital will help students improve their creative ability and achieve self-development of social entrepreneurship (Hamirul et al., 2019). García-Morales and Martín-Rojas (2020) believe that incorporating corporate social responsibility learning into social entrepreneurship courses will promote students’ social entrepreneurship knowledge and skills. What’s more, other scholars have found that the application of social media to social entrepreneurship learning may help students build their own business networks, entrepreneurial knowledge, and sources of financing to start social entrepreneurship businesses (Hamirul et al., 2019). Finally, based on the theory of planned behavior, Chang and Wannamakok (2019) have found that in students who participate in social entrepreneurship learning, their major and subjective behavior norms could have a moderating effect on their social entrepreneurship willingness.

Third, recent research on social entrepreneurship education indicates that cross organizational cooperation is an important trend in the cultivation of social entrepreneurship talents. For example, Bazan et al. (2020) point out that the integration of the external environment and colleges’ initiative play an important role in promoting students’ social entrepreneurship learning. Some scholars also pointed out that the construction of college students’ entrepreneurial environment will help to enhance students’ social entrepreneurial willingness. In addition, García-González and Ramírez-Montoya (2019) believes that the construction of school, government, society, and enterprise – four spiral cooperation projects – will help to cultivate students’ social entrepreneurial and innovation ability. For entrepreneurs in society, Wu and Song (2019) found that social media can promote the learning of entrepreneurship courses, in which trust is an important factor.

#### Traditional Social Entrepreneurship Cultivation Model: “Two-Stage” Type

Nowadays, social entrepreneurship education models in colleges and universities usually adopt the traditional type: “First Class Learning (FCL) + Second Class Learning (SCL) = Landing” (Wang et al., 2019). In this education model, students first acquire an overall framework of the subject through first class learning, then transfer knowledge through activities and competitions in second class learning to develop entrepreneurial qualities, and finally are approved to enter the entrepreneurial park to incubate and launch their start-ups. The advantage of this model is that the teaching system of students is gradual and progressive. The drawback is that the mode is too “textbook” and it is insufficient to stimulate students’ entrepreneurial desire and enthusiasm (Wu et al., 2018). Affected by this, students’ in-depth learning and practical learning of social entrepreneurship will be hindered.

#### New Social Entrepreneurship Cultivation Model:“TSCE”

For the above-mentioned dilemma of talent training, a new social entrepreneurship talent cultivation model may provide us with a solution: the TSCE cultivation model. This model involves teachers and students collaborative innovation or collaborative entrepreneurship. This paper defines TSCE as the following: TSCE is the innovation and inheritance of the traditional cultivation model, which includes the “two stages” of the traditional cultivation model, i.e., “FCL” and “SCL,” More than this, it also has different characteristics in the practical learning of the second class learning. Specifically, in the entrepreneurial practice stage, students and teachers participate in innovation and creation or entrepreneurial preparation. This kind of relationship between teachers and students is a partnership, not necessarily for the purpose of entrepreneurship. It emphasizes the partnership between teachers and students in at least one field of achievement, operation, and resources, and both sides have the same vision in this field. In addition, as a cultivation process, this partnership must exercise or improve the entrepreneurial qualities of students or teachers.

The behavior of TSCE could be measured from three dimensions: achievement dimension, operation dimension, and resource dimension. Achievement dimension focuses on innovation, including the following observation items: Teachers participate in the achievement research and development (R&D) unilaterally; Students then participate in the achievement R&D unilaterally; Both parties then participate in the achievement R&D. Operation dimension focuses on entrepreneurship (including studios launching), and consist of the following observation items: First, teachers do not start businesses, students carry out the business operations; Second, students do not start businesses, teachers carry out the business operations; Third, teachers and students cooperate in business operations; Fourth, teachers and students do not start businesses. Finally, resource dimension includes three sub-dimensions: capital, social relations, and human capital. Capital dimension includes unilateral equity injection, unilateral bond injection, and bilateral equity injection. Human capital includes unilateral (teachers or students) labor input and the labor input of both sides. Social relationship dimension includes unilateral (teachers or students) social relationship input and social relationship input from both sides.

Compared with the traditional cultivation model, TSCE cultivation model has several advantages. Firstly, the model integrates industry and education. In the process of classroom teaching, teachers can take the project as a case which will be put into practice in the following days. In this way, students can feel the atmosphere of entrepreneurship and learn knowledge by conceiving the following practice. Teachers could not only realize their duty of cultivating the talents of relevant fields for their country but can also achieve the goal of scientific research and the transformation of achievements.

Another advantage of TSCE is in its resource sharing and risk co-undertaking. Considering that teachers have a richer social experience, professional knowledge, and extensive relationships, they can provide students with knowledge, financial support, and recommendations for relationship building. Correspondingly, students are full of time and energy and they are willing to promote the completion of the project to enhance their knowledge and ability for their future. They devote to the project together and have the same vision, so they are willing to enjoy the process and face the challenge together.

The final advantage of TSCE is in its strong landing. Teacher–student co-entrepreneurship’s achievements are usually patents and products, which have a mature transformation prospect after the prototype stage, and the team members are familiar with the products, which is conducive to the launch of entrepreneurship. However, it is still unclear whether the incubation effect of the TSCE cultivation model is better than that of the traditional cultivation model. We will use a quantitative method to prove that TSCE is more effective. On the basis of previous research using landing rate, profit amount, subjective evaluation, and other inspection indicators (Shekhar et al., 2018), this paper focuses on the target events of social entrepreneurship studio launch, and examines the differences between the two cultivation models in the incubation effect of social entrepreneurship in colleges and universities through the ratio of students setting up social entrepreneurship studios.

### Application of TSCE in the Incubation of SEO in Colleges and Universities

Teachers and students, as the main body of social entrepreneurship incubation chain in colleges and universities, do not only have a teaching relationship, but also a guiding relationship in the cultivation process, a cooperative relationship in completing projects, and a partnership relationship in innovation and entrepreneurship. They jointly use the entrepreneurship or professional practice platforms provided by the school to produce technology intensive results, some of which can even be transformed, so as to help them to complete their entrepreneurship preparation and start their own businesses.

From the perspective of TSCE cultivation model’s three stages, it usually includes: the conceptualization stage, in which the teacher guides students to carry out professional learning around the project, and revolves around knowledge and skills; the prototype stage, in which the students invest time and energy to assist the teachers in completing the project, and the students realize knowledge transfer, which revolves around innovation; and the commercialization stage, in which products with a market prospect receive the teachers’ capital injection or assistance in operation and the students’ investment of personal human capital and entrepreneurial capital (social resources of relatives and friends) to contribute to the launching of the enterprise. In general, the effectiveness of TSCE is to enhance entrepreneurial learning and entrepreneurial practice through cooperation between teachers and students, and then deepen learning of pre-entrepreneurs in the entrepreneurial preparation stage.

According to the different entrepreneurial visions of teachers and students, TSCE can be divided into three categories: collaborative co-innovation (CCI), collaborative co-entrepreneurship (CCE), and employment co-entrepreneurship (ECE). Collaborative co-innovation is defined as a partnership between teachers and students based on professional topics or projects, with innovation and creation as their common vision, achievement and success as their common interests, and technology creation as their goal. Collaborative co-entrepreneurship is defined in this paper as when both teachers and students take the landing of entrepreneurship as their ultimate goal, always review entrepreneurship preparation progress from the perspective of business owners in school cultivation, and view each other as partners. In this type of TSCE, teachers and students have established a highly trusted partnership. In the type of ECE, either the students or teachers hold the intention to start a business, while the other does not take starting a business as the ultimate goal. In the process of ECE, teachers recruit students to complete their own projects through the incubation process to achieve school assessment. Teachers regard students as immature (technical ability) employees and co-entrepreneurship achievements as their own promotion tools. They are based on the relationship of interest and on the process of supervision and being supervised.

Because this paper focuses on the effect of cultivation models on social entrepreneurship incubation, the CCI cultivation model, which does not take the landing of SEO as its vision, is not calculated in the evaluation type of incubation performance of TSCE. This paper only focuses on the difference of social entrepreneurship incubation performance between CCE and ECE. Based on the theory of social contract (Mehdi et al., 2019), this paper holds that teachers and students who are in a highly trusted partnership are more likely to promote the launch of their SEO and jointly establish social entrepreneurial organizations.

## Data Sources and Research Methods

### National Samples’ Investigation and Preliminary Screening

Basis of Sample Selection. The first principle of selection is the pertinence of the research object. In this paper, we chose graduates who had participated in social entrepreneurship education as the target group. Therefore, we excluded teachers, graduates, and students who did not participate in social entrepreneurship. About 7824 cases of data were obtained after this round of screening. The second selection principle is purpose. As mentioned above, the independent variable of this study is the social entrepreneurship cultivation model (including two levels: traditional cultivation model and TSCE cultivation model). Therefore, the data of 7711 cases were obtained after screening out the students who did not accept the traditional incubation mode. In addition, according to different research purposes, 5258 students who had received the TSCE cultivation model were selected for hypothesis verification.

In the process of data collection, we sent out the electronic questionnaire by its’ uniform resource locator (URI) and quick response link. Questionnaire data were collected and summarized by “Questionnaire Star” (a paid questionnaire tool). The questionnaire data we collected includes three versions: text version, serial number version, and score version.

### Measurement and Scoring of Variables

In this study, we used a self-designed questionnaire. The expert validity of the questionnaire is good, the Cronbach’s α is 0.886. From this point of view, the reliability of the questionnaire is good, and measurement results of core variables are reliable.

The following will explain the connotation, measurement, and calculation of various variables.

Measurement and scoring of research variables. The target variable of our research is whether the students land in the social entrepreneurship studio. This variable is a classified variable, which is sorted by multiple choices to investigate whether students land in the social entrepreneurship studio and its impact on themselves. After the score conversion, it was finally determined that the item of “setting up a social entrepreneurship studio” was recorded as “1” and not as “0.”

Definition of factors. Factors studied in this paper are the social entrepreneurship cultivation model, including two levels: (1) Traditional cultivation model: “FCL + the SCL” mode; and (2) TSCE cultivation model: “FCL + SCL (including TSCE).”

In terms of operational definition, the factor definition of participating in the traditional cultivation model adopts the method of joint verification to select “28. Social entrepreneurship you participated in during school” and “31. The main obstacle for you and your teachers to jointly carry out entrepreneurship projects is” when the respondents choose to participate in “social entrepreneurship lectures, social entrepreneurship activities, social entrepreneurship competitions, social entrepreneurship courses,” and “did not participate in the project which is jointly carried out with teachers” simultaneously, we would believe they only accepted the traditional cultivation model.

The factor of the TSCE is determined by multiple choices. These factors include “ECE” and “CCE.” The scoring item is that “30. The way of cooperation between teachers and students in your entrepreneurial team is”.

The scoring method of “ECE” is to score X points for any X items from option 1 to option 4, and 0 points for no option.

The scoring method of “CCE” is to select option 5 and not select option 1, it is scored as 1 point; if respondents select option 6 and do not select option 5, it is scored as 2 points; if respondents select both (option 5 and option 6), it is scored as 2 points; if respondents select neither (option 5 and option 6), it is scored as 0 points.

## Results

### TSCE Cultivation Model Is More Conducive to Promoting SEO Landing

The purpose of this section is to verify whether the TSCE cultivation model can significantly promote the launch of SEO compared with the traditional cultivation model.

As the target event of this study, “social entrepreneurial organizations landing,” this event is a classified variable. When the dependent variable and independent variables are categorical variables, and the dependent variable isn’t an orderly classified variable, chi-square analysis can be used to verify the effect of independent variables and its effect size.

Therefore, SPSS 20.0 is used for chi-square analysis to investigate the impact of different cultivation models on SEO landing.

It can be seen from Table 1 that, compared with the students who have not accepted the social entrepreneurship education cultivation model in colleges and universities, those students who have participated in the social entrepreneurship education cultivation model are more likely to launch in SEO: *χ^2^* = 5799.512, *p* < 0.001, R_*Zero* model__(__1_._97__%)_ < R_Traditional__C__ultivation model__(__11_._99__%)_, R_Zero model__(__1_._97__%)_ < R _*TSCE* C__ultivation__model__(__77_._94__%)_.

**TABLE 1:** Launch performance of different social entrepreneurship education cultivation models.

	Landing	No landing	Landing ratio	*χ ^2^*	Effect sides
Zero model (zm)	62	3084	1.97%	5799.512***	0.731
Traditional cultivation model	294	2159	11.99%		
TSCE cultivation model	4098	1160	77.94%		

*p < 0.05; **p < 0.01; ***p < 0.001; TSCE: Teacher–student co-entrepreneurship.

In addition, different social entrepreneurship education cultivation models have a significant impact on SEO landing. *χ^2^* = 5799.512, *p* < 0.001, and the effect is large: the effect amount *df*_*min*_ = 292, Cramer = 0.620 > 0.500. Moreover, the promotion effect of TSCE cultivation model on SEO launching is significantly higher than that of the traditional cultivation model (77.94% > 11.99%), R_TSCE c__ultivation__model_ (77.84%) > R_traditional c__ultivation__model_ (11.99%).

The above results show that the existing college social entrepreneurship cultivation model can promote students to land in SEOs. In addition, compared with the traditional cultivation model, the TSCE cultivation model can do better. Therefore, hypothesis 1 can be proven; the promotion effect of the TSCE cultivation model on students’ SEO launching is significantly higher than that of the traditional cultivation model.

### The Incubation Effect of the CCE Cultivation Model on SEO Is Better Than That of the ECE Cultivation Model

The purpose of the study is to define the mechanism of TSCE on SEO landing. Research selects the data of students who accept the TSCE cultivation model.

In order to find out the function route of TSCE on SEO launch, we used the structural equation model to analyze, put the independent variables FCL, SCL, CCE, and ECE into the model, and used RMSEA, CFI, GFI, and CMIN/DF (these indexes are used to determine the goodness of the model by AMOS), and other indicators to observe a fitting indication of the model, with which the model was improved gradually. Finally, we obtained the ideal model through model comparison, residual correction, and path adjustment. The analysis of the data model includes the following steps:

First, we established the original model including only FCL and SCL in traditional mode as the first reference, then we took CCE and ECE as independent variables, and SEO landing as dependent, which was set as the second reference. Next, we put FCL, SCL, CCE, and ECE as independent variables into the same model, as an improved model. Then we added the common variation path between the residual variables according to the AMOS (AMOS: Amos is a software for multivariate analysis using structural equation model) report. Finally, we modified the route between independent variables according to previous operation results, so as to get the final model with good fit. We used AMOS21.0 structural equation model to fit different incubation models. The comparison between five model variable settings and fitting data is as follows (*n* = 5258):

Model 1: Traditional cultivation model (Independent variable: “FCL,” “the SCL”; Dependent variable: social entrepreneurship studio launch).

Model 2: TSCE cultivation model practice period (Independent variable: “CCE”, “ECE”; Dependent variable: social entrepreneurship studio launch).

Model 3: TSCE cultivation model (Independent variable: “FCL,” “SCL,” “CCE,” “ECE”; Dependent variable: social entrepreneurship studio launch).

Model 4: TSCE cultivation model after the correction of residual (Independent variable: “FCL,” “SCL,” “CCE,” “ECE”; Dependent variable: social entrepreneurship studio launch).

Model 5: TSCE cultivation model after path correction (Independent variable: “FCL,” “SCL,”0 “CCE,” “ECE”; Dependent variable: social entrepreneurship studio launch).

The estimated index of each model is shown in Table 2.

**TABLE 2:** Different social entrepreneurship cultivation model’s goodness of fit indexes.

	CMIN/df	RMSEA	GFI	CFI	NFI
Model 1	214.344	0.201	0.974	0.085	0.092
Model 2	490.480	0.305	0.944	0.142	0.145
Model 3	161.815	0.175	0.940	0.092	0.095
Model 4	11.604	0.045	0.999	0.990	0.989
Model 5	4.693	0.022	1.000	0.998	0.997

From Table 2, it can be seen that for graduates who have received social entrepreneurship education in colleges and universities, the data fit best in the TSCE cultivation model of SEO launching, and TSCE more in line with the internal mechanism of social entrepreneurship incubation. With the revision of the model, RMSEA, GFI, CFI, NFI, and CMIN/DF gradually decreased, and the model fit increased, which shows that the model’s compliance with social entrepreneurship incubation mechanism. Finally, we got that the RMESEA of model V is less than 0.05, the CFI, NFI, and GFI are all greater than 0.900, and the sample size of this model is 5258, which belongs to large sample data. The threshold value of the fitting index could be improved CMIN/DF < 5, which shows that model V is well fitted and acceptable.

**FIGURE 2:**
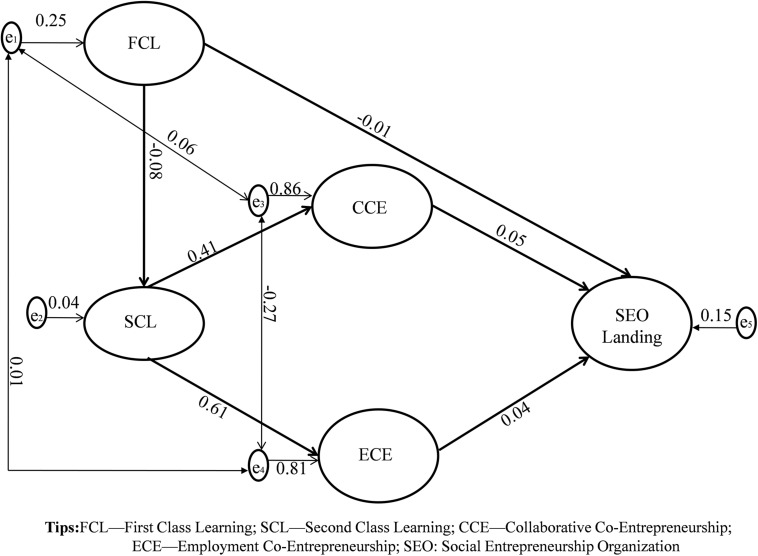
The mechanism of SEO launching under the TSCE cultivation model. The data results are from the analysis of 583 articles with the title of “social entrepreneurship” in the period of 1998–2018 by CiteSpace 5.3.r3 to web of Science (Core Collection).

To further understand the mechanism path of model 5, we will view it through the structural equation diagram as shown in Figure 2.

From Figure 2, it can be seen that for students who have received social entrepreneurship education in colleges and universities, the cultivation model that promotes SEO launch is the TSCE cultivation model. More specifically, there are not only CCE (Co-innovation and Co-operation) β = 0.05, but also ECE (teacher guidance, student entrepreneurship; Teachers’ investment, students’ entrepreneurship; teachers’ R&D, students’ operation; teachers’ operation, students’ participation) β = 0.04, and the CCE promotion effect is greater than ECE [β_*CCE*_ (_0_._05__)_ > β_*ECE(*__0_._04__)_].

Nevertheless, from the traditional cultivation model, FCL not only failed to promote SEO launch, but also inhibited students’ SEO launch β = -0.01. In contrast, students benefited from SCL, which helped to achieve students’ SEO launch. What’s more, TSCE mediates the process from SCL to SEO launch. β_*SCL–CCE–SEO*_ = 0.020. Similarly, ECE also promotes the learning transfer process from the SCL to the SEO launch β_*SCL–ECE–SEO*_ = 0.024.

As mentioned above, hypothesis 2 is proven; the CCE cultivation model is more helpful for students’ SEO launch than the ECE cultivation model.

In general, the results show that the different parts of the traditional cultivation model (“FCL” and “the SCL”) play the reverse function of stimulating SEO launch. In contrast, both kinds of TSCE cultivation model (“CCE” and “ECE”) could help students launch in SEO. In addition, students’ SCL can realize the boosting force of SEO launch through TSCE joined with SCL.

## Discussion

This paper argues that the fundamental driving force of the TSCE cultivation model is the mutual trust between teachers and students, which could promote students’ social entrepreneurship learning and help SEOs to succeed. This shared vision, as an important psychological contract of partners, may enable both partners to achieve greater organizational input and dedication. This will contribute to the realization of common vision and final launch of social entrepreneurship.

### The Realistic Source of CCE Cultivation Model

As the conventional gathering place of technology and knowledge, it is easy to form knowledge collision and innovation in colleges and universities. The trust relationship between teachers and students has been produced since the theoretical teaching period. Trust is one of the preconditions for the effective transfer of knowledge, so it is easier for teachers and students to form an academic community from a professional perspective (Jiang et al., 2019). In the cultivation process of entrepreneurship education, the responsibilities of full-time teachers and entrepreneurship teachers makes them connect with students again (FCL once, SCL twice), which may help students to further deepen the trust relationship in SCL and social entrepreneurship practice. This is the primary advantage of the CCE cultivation model: mutual trust in knowledge.

The second advantage of the CCE cultivation model is vision sharing. When both teachers and students share the same vision, the scope of “public domain,” “responsibility,” and “obligation” recognized by both sides will be highly overlapped. Both of them will be more likely to inspire the initiative to take responsibility and solve the problems encountered from the perspective of team interests. This kind of self-role orientation of protagonist and partner will not only result in the equality of status, but also help to achieve a high level of trust, and ultimately jointly create an efficient team that dares to start a business, is able to start a business, and is willing to start a business, and could finally achieve SEO launch.

What needs to be explained here is that the common vision defined in this paper does not only refer to the entrepreneurial vision. For the conceptualization stage and prototype stage, teachers and students may have the same expectation for technological innovation or academic contribution. But it is undeniable that the trust atmosphere based on knowledge transfer and knowledge sharing is an important guarantee for the efficient operation of the team in this stage. At the same time, we should be alert to preventing the social exclusion and devaluation caused by educational background, achievements, and reputation in the process of research and development, which will lead to the rupture of the trust relationship, and lead to the decline of organizational effectiveness.

### The Practical Malpractice Reflected by ECE Cultivation Model

Because of the utilitarian principles of higher education in China, teachers are more willing to produce results than to implement them. In this regard, students and teachers form a potential employment relationship through entrepreneurial competition, rather than a real partnership (students get awards for academic promotion in the form of job searches and teachers get awards for professional promotion). This also explains why in Figure 2, the regression coefficient of the ECE path is smaller than that of the CCE path (β_*CCE–SEO*_ = 0.05, β_*ECE–SEO*_ = 0.04, β_*ECE–SEO*_ < β_*CCE–SEO*_). This proves the conclusion from one side, which is that when teachers and students share a common vision of co-entrepreneurship or co-innovation, students can be more inspired to situate themselves in social entrepreneurial organizations.

To explore the reasons behind it, such a phenomenon may originate from the deviation of responsibility category and self-role positioning between teachers and students. When both teachers and students hold different vision goals, the scope of “public domain” and “responsibility obligation” recognized by both sides is difficult to achieve a high degree of coincidence. Both of them are more likely to avoid responsibility, escape from problems they meet, and take care of their own interests. This role orientation of individual interests is driven by interests, forming a team with low trust, weak effectiveness, and difficulty in SEO launching.

### Implication

In this paper, the establishment of SEO is greatly promoted by TSCE and the common vision, which is fundamentally due to the high organizational commitment and organizational dedication, which comes from the high trust between team members. Therefore, for teachers or students who are interested in innovation or entrepreneurship in groups, to ensure good operation performance of entrepreneurial teams, we need to ensure this high level of trust in the team. Specifically, this paper believes that it can be fulfilled from two aspects: team vision and team trust.

The assurance of team vision is reflected in the selection of team members. It includes two recruitment requirements. One requirement is to choose members with the same entrepreneurial vision. Team creators should ensure the prospective team members are willing to participate in innovation or entrepreneurship and pursue the same goals, and they also need to combine pre-members’ career development plans in school to determine whether their entrepreneurial goals and its’ realization degree are consistent or similar with the creator’s. From the perspective of reality control, it is usually based on the experience of the recruiter, combined with the characteristics of the recruiter, such as grade, graduation destination, character (recruiting students), title, career dilemma, entrepreneurial willingness (recruiting teachers), etc., to organize and build a team.

The other requirement is to ensure that the team members selected have personality traits such as high organizational dedication or high organizational citizenship behavior. Facing the uncertain process and vague goal of innovation or entrepreneurship, team members need to invest a lot of time and energy. They cannot give up and complain easily because of short-term difficulties. They need to have the dedication to put the team interests above their own. The measurement of these personality factors can be realized through existing questionnaires.

The assurance of team trust refers to maintaining a high level of trust among team members in the innovation or entrepreneurship process. To achieve a high trust level, there are also two solutions which can be used to fill the trust gap and establish the organizational contract with the team interests’ priority. In the communication network of the whole team, the boundless circulation of trust in the work field could ensure team members communicate with each other in time on unexpected problems and entrepreneurial difficulties, and work together to solve and invest. In practice, we can offer items like “who would you complain to when you meet difficulties?” or “who would you ask for help when you solve difficulties?” to use social network questionnaires to detect the trust gap (i.e., isolated or one-way links) in team emotional networks and assistant networks, so as to fix trust problems and repair separable relationships.

The second solution is to establish organizational contracts with the team interests’ priority. We suggest that psychological contracts of trust among team members should be guaranteed by organizing written contracts. Considering this, we are most concerned about the distribution of team benefits. From the perspective of necessity, early discussion or implementation of interest distribution may lead to uneven interest distribution and damage the trust among members. Therefore, the benefit distribution should be carried out after the team goal is achieved. Secondly, we emphasize that the team benefits are distributed first, and it is communicated to the members of the organization that the achievement of individual value depends on the realization of other members’ value, so that they pay more attention to other members’ demands and communicate with other members. This empathy is conducive to bringing about deep trust. In terms of practice, we could define the distribution period, principles, and prohibitions of team operation by means of legal contracts.

From a theoretical point of view, the theoretical basis of this study is social contract theory. The subject of this theory was developed from the early state to the later enterprise and informal organization. The theory emphasizes that each side of the contract should implement their own obligations, which is also an important process to ensure the freedom of the other side. On the basis of previous studies, this study confirms that the binding force of social contracts still exists in public education organizations such as Chinese colleges and universities, and proves that its binding force on implicit agreements (common vision, priority of team interests) will affect the trust relationship, while those who does not obey the implicit agreements will make their partners uncooperative or negatively cooperative (it could be reflected by effectiveness differences between CCE and ECE).

Generally speaking, this paper hopes to find and describe a new and effective entrepreneurship education cultivation model. We confirmed its effect through quantitative research methods and called for the promotion of the TSCE cultivation model. We hope that the TSCE cultivation model could help more teachers and students to carry out social entrepreneurship learning and improve their social entrepreneurship capability, so as to realize the more adequate preparation for future opportunities for these pre-social entrepreneurs.

## Conclusion

In the cultivation process of stimulating students to establish SEOs, the TSCE cultivation model is more effective than the cultivation model; the CCE cultivation model is more conducive to helping students to land in SEOs than ECE cultivation models. The ECE cultivation model is better than the CCE cultivation model in integrating entrepreneurship practice of the traditional cultivation model learning.

## Limitation

Although this research has a wide range of samples, it only uses the method of post evaluation to make a horizontal comparison. We suggest that the follow-up study can adopt the tracking method to compare the change of students’ social entrepreneurship willingness before and after their participation in the TSCE cultivation model.

The target variable selected in our study is whether to establish a social entrepreneurship studio. This indicator is relatively single. We hope that the follow-up study should be carried out on this basis, combined with subjective indicators, such as satisfaction of social entrepreneurship education in colleges and universities, to conduct a multi-dimensional verification. This should be done in order to investigate the role of different cultivation models in stimulating students’ Social Entrepreneurship comprehensively.

In our study, only graduates were selected for investigation, and it is suggested that future research can include current students in the investigation. This would enable the detection and summarization of the possible new social entrepreneurship cultivation model in time, to compare and analyze with the existing mode, providing a more timely and targeted reference for social entrepreneurship incubation in colleges and universities.

## Data Availability Statement

The raw data supporting the conclusions of this article will be made available by the authors, without undue reservation.

## Ethics Statement

The studies involving human participants were reviewed and approved by Ethics Committee of Wenzhou Medical University. The patients/participants provided their written informed consent to participate in this study.

## Author Contributions

JD finished the work of literature reading, research design, and data analysis and completed the writing of the draft, the second draft, and the revision of the final draft. In the data collection phase, JD and GH completed the preparation of the questionnaire. ZH provided guidance for the improvement of the questionnaire content, assisted JD to complete the preliminary test and retest of the questionnaire, and provided the revision of the first draft and the revision of the second draft. ZH and GH provided assistance in research funds and resources, including questionnaire printing, investigator charge, etc. and completed the revision of the final draft. All authors contributed to the article and approved the submitted version.

## Conflict of Interest

The authors declare that the research was conducted in the absence of any commercial or financial relationships that could be construed as a potential conflict of interest.
